# The North American Repository for Archaeological Isotopes

**DOI:** 10.1038/s41597-024-04175-2

**Published:** 2025-01-11

**Authors:** Traci N. Billings, Erin Scott, Carlo Cocozza, Sean Hixon, Nicole Boivin, Patrick Roberts, Robert N. Spengler, Ricardo Fernandes

**Affiliations:** 1 Department of Archaeology, Max Planck Institute of Geoanthropology, Jena, 07745 Germany; 2 Institute for Prehistoric and Protohistoric Archaeology, Christian-Albrechts-University of Kiel, Kiel, 24118 Germany; 3 Domestication and Anthropogenic Evolution Research Group, Max Planck Institute of Geoanthropology, Jena, 07745 Germany; 4 Mediterranean bioArchaeological Research Advances (MAReA) centre, Università degli studi della Campania “Luigi Vanvitelli”, Caserta, 81100 Italy; 5 ArchaeoBioCenter (ABC), Ludwig-Maximilians-Universität München, München, 80539 Germany; 6 Department of Integrative Biology, Oregon State University, Corvallis, OR 97331 USA; 7 School of Social Science, The University of Queensland, Brisbane, QLD 4071 Australia; 8 Griffith Sciences, Griffith University, Nathan, QLD 4111 Australia; 9 isoTROPIC Research Group, Max Planck Institute of Geoanthropology, Jena, 07745 Germany; 10 Department of Bioarchaeology, Faculty of Archaeology, University of Warsaw, Warszawa, 00-927 Poland; 11 Climate Change and History Research Initiative, Princeton University, Princeton, NJ 08544 USA; 12 Faculty of Arts, Masaryk University, Brno, 602 00 Czech Republic

**Keywords:** Palaeoecology, Agriculture

## Abstract

Here, we present the *North American Repository for Archaeological Isotopes (NARIA)*, the largest open-access compilation of previously reported isotopic measurements (n = 28,374) from bioarchaeological samples in North America (i.e., Canada, Greenland, Mexico, and the United States of America) covering a time-frame of more than 12,000 years. This database consists of stable (δ^13^C, δ^15^N, δ^18^O) and radiogenic (^87^Sr/^86^Sr) isotope measurements from archaeological human, animal, and plant sources and their corresponding contextual information (e.g., location, chronology, cultural affiliation, etc.). This synthesis of isotopic measurements and other forms of data presents significant research potential for investigating past human lifeways, particularly in the realms of paleomobility, paleoenvironment, and paleodiet. Additionally, it serves to pinpoint spatial and temporal data gaps, offering valuable insights for future research directions.

## Background & Summary

Although stable isotope analysis has a rich legacy of application in the fields of environmental science, chemistry, and geology^1–4^, it was Vogel and van der Merwe’s seminal research in the 1970s that saw the first use of stable isotopes to answer archaeological research questions in prehistoric North America^5,6^. Vogel and van der Merwe used stable carbon isotope analysis to investigate the consumption of maize in a prehistoric population from the northeastern woodlands (i.e., modern day New York state^5,6^). This research was based on the premise that stable carbon isotope measurements from C_3_ plants (most trees, shrubs, and grasses found in temperate regions) and C_4_ plants (tropical grasses from hot or arid regions) have distinct, identifiable ranges^7,8^. Since a plant’s photosynthetic pathway is determined by how it fixes atmospheric CO_2_^9,10^, the input of maize (a C_4_ plant) is isotopically distinct from a predominately C_3_ resource base in the diet of ancient humans. This basic premise inspired and laid the foundation for more than four decades of isotope research in North American archaeology. Much early research was focused on paleodietary reconstruction specifically aimed at understanding the spread and consumption of maize and its connections to cultural, social, and economic changes among prehistoric populations (e.g.^11–15^). Other applications included exploring relative human reliance on marine and terrestrial resources (e.g.^16–18^), and relative plant vs. animal protein intake (or ‘trophic level’^19,20^, other significant early studies in this regard outside North America included^21,22^). While most of these studies focused on δ^13^C and δ^15^N measurements, researchers also began to investigate the potential of ^87^Sr/^86^Sr isotopes analysis for understanding diet and mobility^23–26^.

During this period of early exploration, questions of dietary routing into different tissues, as well as fractionation effects between diet and consumer, soon emerged in discussions of isotopic interpretation (e.g.^19,27–29^). There was also an increasing interest in exploring the preservation and reliability of isotopes from archaeological material that had likely undergone diagenetic alteration (e.g., affected by temperature, moisture, pH, microbial attack, and deposition time^30–34^). This prompted a series of experimental feeding studies^27,35,36^ that have continued to date (e.g.^37^). Application of strontium isotope analysis to archaeological contexts as a tracer of geological location during tissue formation also emerged at this time^23–26^ with experimental studies designed to reveal how strontium becomes a part of the mineral portion of bioapatite (e.g.^38^) and how it exchanges between tissues and the burial environment (e.g.^39^). Over the past two decades, isotope research has increasingly been applied to investigate a diverse range of topics in North American archaeology such as, paleodiet (e.g.^40–45^), human mobility (e.g.^26,46,47^), animal management and exchange (e.g.^48–50^), the use of analyses of dogs as proxies for human diets (e.g.^51–54^), animal migration patterns (e.g.^55–57^), and paleoenvironmental reconstruction (e.g.^58^).

Advances both in technology (e.g., continuous flow mass spectrometry) and methods (e.g., sample pretreatment, data calibration) have also made it easier, faster, and less expensive to apply isotope analyses to tissues from humans, animals, and plants as proxies to reconstruct past food systems, environments, climates, and mobility patterns^59–62^. Mixing models^63^ and Bayesian mixing models (e.g.^64^) have been applied in several regional studies to try to parse out the contribution of different types of food sources to prehistoric consumer diets (e.g.^65–69^). Today, bulk and compound specific stable isotope analysis continues to be applied to human, animal, and plant samples to answer a variety of archaeological research questions in North American contexts across space and time (e.g.^70–74^).

With the growing application of stable isotope studies to address topics surrounding past diet, mobility, and ecology in the past, there is simultaneously an increasing need for more representative datasets (i.e., larger datasets with contextual information) and more standardized reporting to best practices, meaning the inclusion of data with which other researchers can assess the quality of the data provided (e.g., %C, %N, C/N, collagen weight^61,62,75^). Moreover, the need for more contemporary and locally-relevant baseline data to enhance our understanding of isotope proxies is becoming increasingly apparent^62^. Large, well curated isotopic datasets, which include contextual information and quality control data, are particularly suited to address these issues. Big isotope data also allows archaeologists to explore questions concerning diet, environment, and mobility at different spatial and temporal scales and to identify potential gaps in research that can be targeted for future exploration, as demonstrated by recent publications^76–78^. However, despite the relatively large amount of available isotopic measurements from bioarchaeological contexts in North America, there remains no open-access centralized repository of such data.

**Fig. 1:**
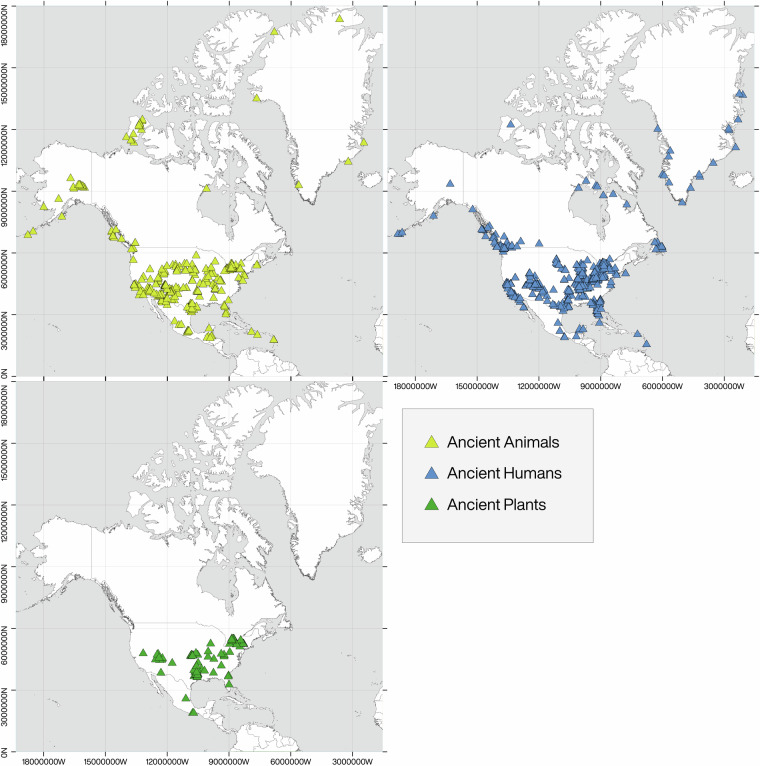
Spatial distribution of archaeological human, faunal, and floral stable isotope values in North America currently compiled within NARIA. Geographic Coordinate System: World Geodetic System 1984 (WGS84). Figure Layout: Hans-Georg Sell, for the Max Planck Institute of Geoanthropology.

**Fig. 2:**
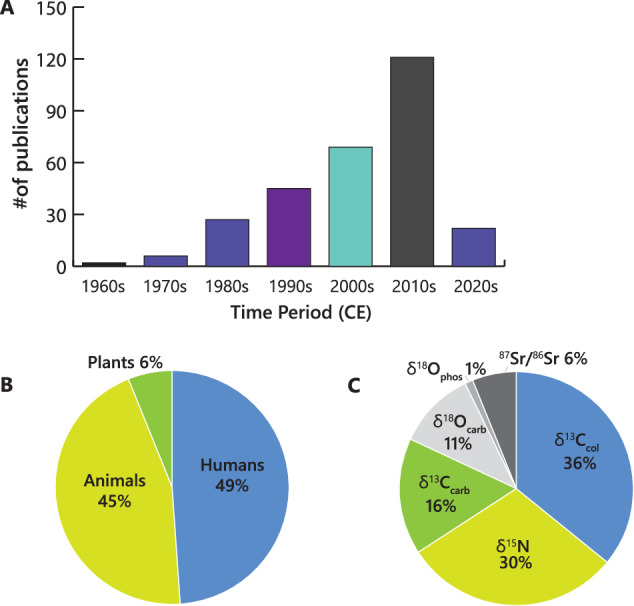
Overall summary of the data. (**A**) number of publications vs. year, (**B**) percent of animal, human, and plant samples in the repository, (**C**) percentages of different types of isotope data that can be found in NARIA.

**Fig. 3:**
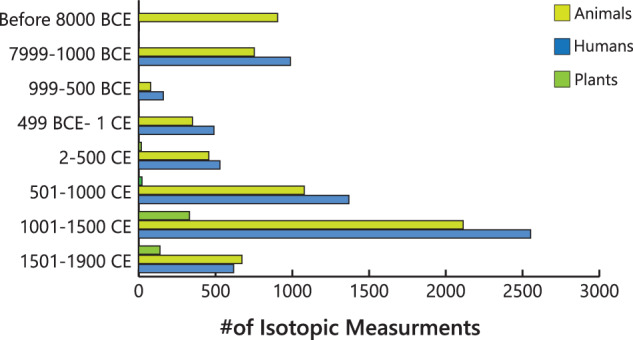
Number of measurements through time. The periods are divided: the Paleoindian period (~18,000–8000 B.C.E.), the Archaic (7999–1000 B.C.E.), then every 500 years starting at 999 B.C.E. until 1900 C.E.

Here, we present, the *North American Repository for Archaeological Isotopes (NARIA*; 10.48493/hnhn-7158^79^), a partner of the IsoMemo network of autonomous databases. *NARIA* is a research resource to be used to further develop isotopic research in archaeology. It consists of a compilation of previously reported isotopic measurements (n = 28,374) on bone collagen, bone bioapatite, tooth enamel bioapatite, and tooth dentin collagen from humans and animals, and tissue from plants found in archaeological contexts in North America (i.e., Canada, Greenland, Mexico, and the United States of America; Fig. 1). The isotope data were compiled from previously published material spanning almost five decades of research. The human and animal isotope values and corresponding metadata collected are from archaeological sources. The plant isotope data and metadata represent both archaeological and modern sources, although emphasis was placed on the former. A general overview of the data which outlines the number of isotopic measurements through time, the proportion of data from each type of stable isotope, the proportion of data from each source material (i.e., human, plant, animal), and a breakdown of references used in this research presenting the number of publications per year can be found in Figs. 2, 3.

## Methods

Previously published isotope measurements and corresponding contextual information from prehistoric North American human, animal, and plant samples were systematically collected from available publications found on Google Scholar and similar web search engines through December 2023. Several topic specific search terms were used for locating articles (e.g., maize, isotopes, North America). Bibliographies were also searched for relevant source material. An effort was made to find the original publication source for all data. However, if this was not available but the data could be found in a secondary source, the data were still collected, and this was noted. When accessible, grey literature (e.g., theses, unpublished reports) was included. Sometimes isotope measurement data were published but not reported in a form that could be reliably recorded (e.g., if the records were only reported in a low-resolution graph form). In these cases, data were not recorded. While a rigorous search was conducted to find all available material, it is likely that some publications or other resources may have been missed, especially as we only performed searches of material published in English. To offset these concerns the datasets have been setup so that new material can be easily added in the future.

The data were recorded as reported in the original publication. If for some reason the information was changed, an explanation has been offered in the *Comments* column. Sometimes it was necessary to standardize the data to allow for analysis (e.g., the ‘curated age category’ of an individual was recorded following the categories given in^80^). If information was not reported or not known, the corresponding cell in the dataset was left blank.

Metadata were distributed across three separate tables, one for each type of source material (i.e., human, animal, plant). These tables were tailored to the source material but were modelled with reference to best practices described in Roberts *et al*.^61^ and follow a similar organization to other databases within the IsoMemo network (https://isomemoapp.com/^76–78^). The structure of this metadata is presented below and further described in Supplementary Tables 1–9. The rows in each Excel file correspond to individual reported measurements. The columns correspond to different metadata categories which have been grouped thematically using subheadings and explained below. This information is also available within each dataset in the form of a table under the tab ‘Metadata’.

### User information

A unique identifier (*Entry ID*) was given to each isotopic measurement collected to ensure our ability to adequately index, organize, and curate the dataset. The submitter (*ID user*) was also recorded.

### Sample identifiers and descriptive information

In addition to the unique identifier (*Entry ID*), the sample identification (*Sample ID*), and the individual identification (*Individual ID*) were collected. Sometimes these fields are reported as the same, because that is how they were referred to in the original publication. When available, descriptive information about the sample was collected, including its taxonomy (*Taxon, Taxon family name*), common name (*Common name*), the general animal category that it is assigned (*General category*), demographic information (*Sex*, *Age category of individual, Min. age individual (yrs), Max. age individual (yrs)*), and information about the sample material (*Osteological sample, and Osteological sample part*). For animal entries, an additional column (i.e., *Taxon family name*) was added. A brief description of each of these metadata categories can be found in Supplementary Table 2. Slightly different metadata categories were used for entries of plant data (e.g., photosynthetic pathway, plant part; Supplementary Table 3).

### Sample contextual information

Site information including the *Site name*, *Site description*, *Context description*, *Context ID*, *Altitude*, *Latitude*, and *Longitude* were recorded. Descriptions of each of these fields can be found in Supplementary Table 4. The location of the site is particularly important for being able to spatially place isotopic measurements. However, this information is not always provided in a straightforward manner in source publication and reports. Additionally, in some cases, precise coordinates are deliberately omitted because of heritage protection laws. If no coordinates were given for a site’s location, then the maps and descriptions provided in the original publication were used to estimate its location on Google Maps. If no coordinates, map, or description of the site were given, or if these were vague or incomplete, an effort was made to find other sources which would have reported this information. If another source was used to find the site’s location, it was noted within the *Comments*. To further clarify the estimated entries, two columns, *Exact Site Location* and *Radius (km)*, were added (descriptions provided in Supplementary Table 4). The *Radius (km)* column allows the recorder to add an estimate of uncertainty to the decimal coordinate values. For example, if the location is estimated as x, y with a radius of 10 km based on the available evidence, the recorder is indicating that the site is within 10 km of this location. Latitude and Longitude coordinates were recorded in decimal degrees and are with reference to World Geodetic System 1984 (WGS84).

### Sample dating information

To be able to temporally locate the isotope measurements, dating information (i.e., *Min year (BCE/CE)*, *Max year (BCE/CE), Dating method*, ^14^*C*, ^14^*C unc*., and *Period tags*) for each sample was collected where applicable and available. A brief description of each of these fields can be found in Supplementary Table 5. Determining the chronology associated with the source material was sometimes challenging. We adopted a hierarchical approach to assigning chronology. If a specimen had been directly radiocarbon dated, its uncalibrated dates and corresponding information were recorded. If a specimen was not directly dated, it was dated by association to other artifacts (e.g., via pottery typology, lithic typology, stratigraphic information). If radiocarbon dates were available for an artifact associated to the sample, then this date was used. Often specimens were not directly dated but placed within a cultural period (e.g., Archaic, Mississippian) corresponding to other material found with them or a site’s general date. If the literature only reported a time period or cultural association for the site, it often decreased the precision to which specimens could be dated. Other challenges that arose included a lack of consensus on a specific time frame for a cultural period, a lack of clarity in reporting which material belonged to which period, and/or a lack of clarity concerning the general stratigraphy. Information pertaining to how the date for each specimen was recorded is listed in the “*Dating methods*” column. Any inconsistencies or challenges associated with dating a specimen (e.g., date was given as cultural period but the time range for the specific cultural period is debated, etc) are noted in the “*Comments*”.

### Bibliographic information

Each isotopic measurement is linked to the original publication in which it was reported through four metadata categories including *Reference*, *Link*, *DOI*, and *Publication year*. The reference is given in Harvard Reference Style. When available a link to the original source material (*Link*) and the data object identifier (*DOI*) are included. The date of the publication (*Publication year*) is given as a year (CE). A column that indicates if data were taken from another database or a review paper (*Compilation study*) has also been included.

### Stable isotope measurements

Following best practices for reporting isotopic measurements^61,75^, δ^13^C and δ^15^N bulk collagen, δ^13^C and δ^18^O bioapatite carbonate, δ^18^O bioapatite phosphate, and ^87^Sr/^86^Sr bioapatite stable isotope values were collected. Different types of isotope measurements require different information to be reported so that they may be assessed for reliability. For stable carbon and nitrogen bulk isotope measurements the following categories of data were targeted: *Measurement ID collagen δ*^13^*C & δ*^15^*N*, *Lab name collagen δ*^13^*C & δ*^15^*N, Nr. of measured samples (collagen δ*^13^*C & δ*^15^*N)*, *Sample preparation collagen δ*^13^*C & δ*^15^*N*, *Analysis collagen δ*^13^*C & δ*^15^*N*, *IRMS collagen δ*^13^*C*, *IRMS collagen δ*^13^*C unc*., *collagen δ*^15^*N*, *collagen δ*^15^*N unc*., *Collagen yield*, *%C*, *%N*, and *Atomic C:N ratio*. For δ^13^C and δ^18^O bioapatite carbonate isotope measurements, the following categories were included: *Measurement ID carbonate δ*^13^*C & δ*^18^*O*, *Lab name carbonate* δ^13^C & δ^18^O, *Nr. of measured samples (carbonate δ*^13^*C & δ*^18^*O)*, *Sample preparation carbonate δ*^13^*C & δ*^18^*O*, *Analysis carbonate δ*^13^*C & δ*^18^*O*, *Carbonate δ*^13^*C*, *Carbonate δ*^13^*C unc*., *Carbonate δ*^18^*O*, *Carbonate δ*^18^*O unc*., and *Reporting standard for Carbonate δ*^18^*O*. For δ^18^O phosphate isotope measurements the following categories were collected, *Measurement ID phosphate δ*^18^*O*, *Lab name phosphate δ*^18^*O*, *Nr. of measured samples (phosphate δ*^18^*O)*, *Sample preparation phosphate δ*^18^*O*, *Analysis phosphate δ*^18^*O*, *Phosphate δ* ^18^*O, Phosphate δ* ^18^*O unc*., and *Reporting standard for Phosphate δ*^18^*O*. For the ^87^*Sr/*^86^Sr bioapatite isotope measurements the following categories were added: *Measurement ID* ^87^*Sr/*^86^*Sr*, *Lab name* ^87^*Sr/*^86^*Sr*, *Nr. of measurements* ^87^*Sr/*^86^*Sr*, *Sample preparation* ^87^*Sr/*^86^*Sr*, *Analysis* ^87^*Sr/*^86^*Sr*, ^87^*Sr/*^86^*Sr*, ^87^*Sr/*^86^*Sr unc*., and *Sr ppm*. Descriptions of these metadata categories can be found in Supplementary Tables 6–9, respectively. Note that compound specific isotope measurements are not included in these datasets.

## Data Records

Overall, the *NARIA* contains 28,374 isotopic measurements from 14,313 individual records and consists of primary, secondary, processed, and interpreted data compiled from existing publications and grey material. The datasets are presented as three Excel (.xlsx) files and three CSV files. Additionally, the corresponding references are provided in a separate text file in Word (.doc) and RTF formats. The datasets are in English, except for the taxonomic designations which are given in Latin. The datasets are available via Pandora within the repository of the *North American Repository for Archaeological Isotopes (NARIA)* data community: https://pandoradata.earth/dataset/naria-datasets with the 10.48493/hnhn-7158^79^ under a Creative Commons Attribution license. NARIA is also a member of the IsoMemo network of isotopic databases (https://isomemoapp.com/). A list of individual dataset files and brief description are provided below. Human data ranging from 7050 B.C.E. to 1880 C.E. was collected, while the fauna data span millions of years. Plant data comes from samples taken from archaeological sites since 1300 B.C.E. but also includes more recent measurements on herbarium material and modern plants for comparison (although modern plant measurements require that researcher apply a *Suess effect* correction). As seen in Fig. 1, a majority of the data comes from human and animal isotope measurements.

**Fig. 4:**
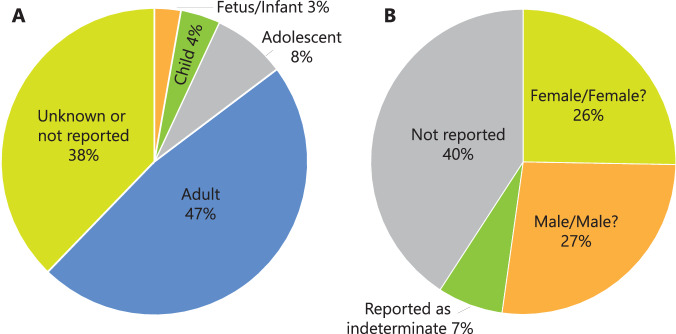
Percentages of (**A**) age categories and (**B**) sex designations collected in human dataset.

**Fig. 5:**
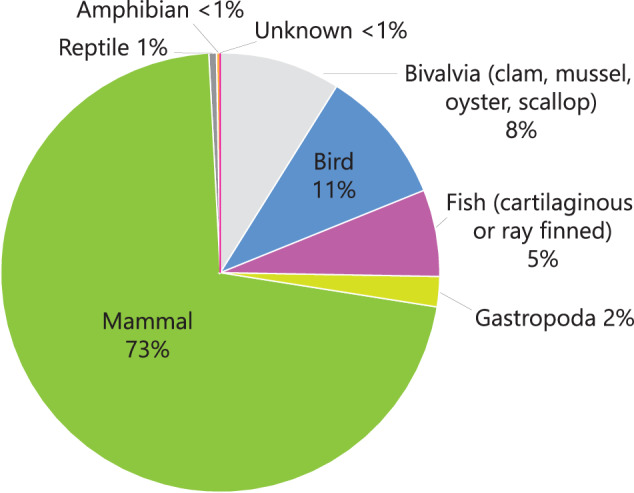
Percentages of the different types of animals present in the fauna dataset.

The *North_American_archaeological_human_isotopes* dataset contains 6,724 individual entries of archaeological human bone and teeth samples from ancient North American populations. A simplified summary of the archaeological contextual information from this data set can be found in Fig. 4A,B. The *North_American_archaeological_fauna_isotopes* dataset contains 6,463 individual entries of archaeological animal bone and teeth from ancient North American populations. A summary of the proportion of each general animal type, as well as a list of family taxa and corresponding number of measurements, can be found in Fig. 5 and Supplementary Table 10, respectively. Mammals are the predominant general animal type in the dataset. Samples from Cervidae, Bovidae, and Canidae are the most frequently recorded family taxa.

The *North_American_modern_and_archaeological_flora_isotopes* dataset contains 1,126 individual entries of archaeological and modern plant remains from North America. Emphasis was placed on collecting isotope values from ancient plant material. Modern plant values associated with ecological baseline studies were often reported in the archaeological literature. We tried to incorporate, when time allowed, these modern plant measurements, but they were not collected in a systematic manner, though this work is planned for the future. Most of the ancient plant material comes from isotope measurements on maize (*Zea mays*). Only δ^13^C and δ^15^N bulk values of plants have currently been collected but the dataset framework is set up for other types of isotope analyses as well. The *North_American_isotopes_bibliography* includes a reference list to original publications from which the data were compiled. Each dataset file also includes a reference for each individual measurement to ensure that the original publication has been cited.

## Technical Validation

The ability to adequately assess and ensure the reliability of isotopic measurements from archaeological material is a major source of concern in the field of isotopic research, given the likelihood that ancient samples have undergone diagenesis (i.e., been altered by post-mortem and post-depositional processes^32,60^). Much research has been conducted to address the influence of diagenetic processes on isotopic measurements from ancient material (e.g.^31–34^). To allow researchers to check the reliability of isotopic measurements and assess preservation, the standard parameters (i.e., %C, %N, collagen yield^81^ and the C:N atomic ratios^82^) were recorded when provided in the original publications. C:N atomic ratios (atomic *C:N ratio*) are a crucial diagenetic test and allow researchers to assess the quality of data reported. However, we did not apply any criteria for exclusion of samples in this regard as the recommended ranges and the criteria themselves are not universal (e.g.^81–83^). Isotopic measurements were also collected when preservation criteria were not reported. By not applying any exclusion criteria, we allow for researchers to adjust their data selection to align with their specific research objectives. The inclusion of samples which fall outside recommended ratios for these criteria also present an opportunity for future studies on sample preservation.

The improvements in methods (e.g., pretreatment and calibration) for measuring stable isotopes over the last five decades also presents a significant challenge when determining the suitability of comparisons^61,84^. For example, stable carbon isotopic measurements reported from different types of analysis (i.e., measurements on isotope ratio mass spectrometer- IRMS vs. accelerator mass spectrometer- AMS) are sometimes not readily cross comparable^85,86^ especially, when necessary, standards are not run in the process of AMS. Most studies reporting isotopic measurements come from studies utilizing an IRMS. Entries from isotopic measurements performed on an AMS in the plant dataset were noted in the *Comments* column and a separate field, *AMS*, which can easily be filtered. To allow researchers to assess the appropriateness of comparisons, descriptions of sample pretreatment, analyses, uncertainties, and standard/reference material were collected when available.

## Usage Notes

The compilation of this data into a usable dataset was challenging because of the lack of standardization in reporting practices of radiocarbon and isotopic data over the last 47 years. Several recent articles have discussed the need for clearer standards in reporting isotopic data in archaeological studies (e.g.^61,62,75,87^). Proper reporting is essential for assessing the quality of the data and associated interpretations. To facilitate future use, the metadata categories of our database were designed to include recent standardized reporting categories in line with current best practices. Isotopic measurements and their corresponding contextual information were collected as they were reported in the original publications unless otherwise noted. In some cases, the data needed to be curated to facilitate standardization of the dataset (e.g., spelling of site names, common name for bone elements, reported age categories, etc.).

Spatial and temporal resolution is not consistent across the datasets. Much of the data lacks precise chronology. Direct radiocarbon dates from source material were not always reported, and, when reported, frequently omit crucial assessment parameters (e.g., lab codes, calibration software and specifications, whether the date was conventional or AMS, etc). The dates provided for samples in publications are often from associated material, overall site occupation dates, or cultural period estimations. The availability and accuracy of reported locational data is also inconsistent across the dataset. Many publications do not report exact geographical coordinates and instead provide only a map and/or description of the site. These limitations should be kept in mind when using these datasets.

Data was checked for duplicates and duplicates were deleted. Each row corresponds to an individual entry with a unique identifier (*Entry ID*). In most cases, this pertains to a single individual. However, entries can also encompass averages derived from isotopic measurements conducted on multiple individuals or replicates of isotopic measurements performed on the same individual. To make these entries easily identifiable, measurements pertaining to averages were noted in the *Sample description* and in the *Number of measurements* columns of the type of isotope being reported. If multiple measurements were taken on the same individual but reported in different publications or in cases where replicate or sequential measurements were provided, that individual may have multiple entries. This information has been indicated in the *Comments*. Any other discrepancies which deviated with the methods for collecting data described in the Methods section above were included in the *Comments* to facilitate future data use.

All data, excluding exact site coordinate data, is publicly accessible via the Pandora platform. The site coordinate data has been withheld to protect archaeological sites from damage but are available to verified academic researchers upon email request. Users are encouraged to read the descriptions within the *Comments* column to know if there are any issues concerning the data that they should be aware of when using it for analyses. Data can easily be filtered to fit users’ requirements.

**Fig. 6:**
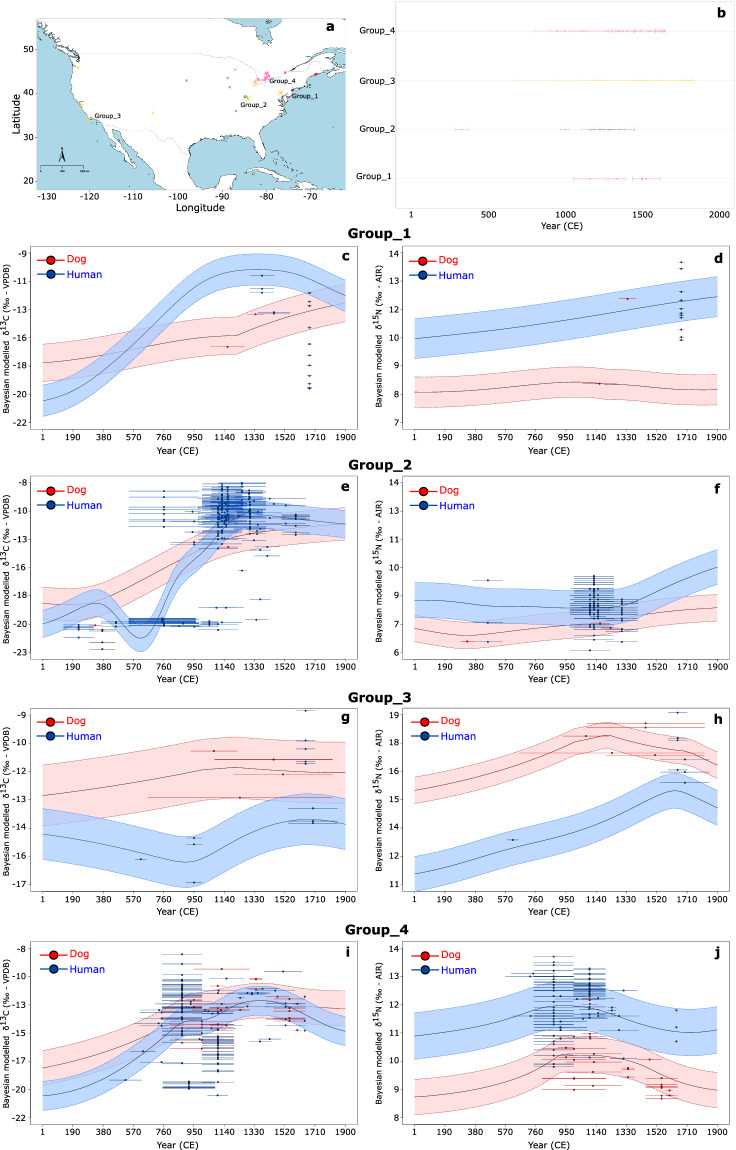
(**a**) Distribution of dog isotopic data and identified clusters; (**b**) temporal coverage of dog isotopic data for each cluster. Horizontal lines for each point represent temporal range associated to each sample. (**c**–**j**) modelled temporal trend for mean δ^13^C_coll_ and δ^15^N_coll_ values at the centroid of each cluster location. The coloured bands around each mean line corresponds to a 95% credible interval. The points shown correspond to isotopic measurements located within a 250 km radius of each centroid and the respective horizontal lines are the temporal ranges associated to each point.

To briefly illustrate NARIA’s research potential we have included here two brief modelling examples. Figure 6 plots Bayesian temporal estimates of δ^13^C_coll_ and δ^15^N_coll_ on adult human and dog bone collagen at four separate locations. These locations were selected as centroids of four identified clusters according to the distribution of dog isotopic data. The human modelled results can be employed to study agricultural practices and overall show a temporal rise in δ^13^C_coll_ corresponding to the intensification of maize agriculture followed by a decrease ensuing European colonization. Exemplifying the study of human-animal relationships, the comparison of human vs. dog δ^13^C_coll_ and δ^15^N_coll_ temporal plots shows that these follow similar trends and attest for the long-standing close bond between humans and dogs.

**Fig. 7:**
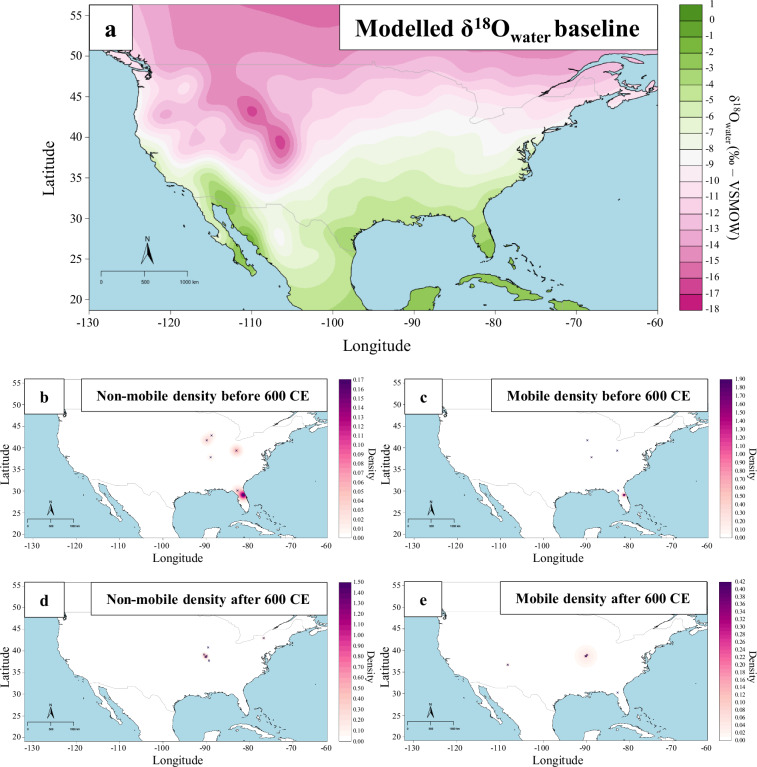
(**a**) Bayesian estimate of the distribution of modern water δ^18^O; (**b**–**e**) kernel density and distribution of individuals identified as mobile or non-mobile before and after 600 CE relative to the modern water δ^18^O baseline.

Another research application of NARIA is the study of past spatial mobility patterns in both humans and animals. Here (Fig. 7), we defined mobile human individuals as those with estimates of ingested water δ^18^O (estimated from human tooth δ^18^O measurements) beyond a certain range of the water baseline value δ^18^O at the respective burial place. In a comparison of human samples dating before and after 600 CE it is possible to identify mobile individuals for both periods. However, the data is too scarce to propose clear temporal patterns in human mobility. Both in this example and that shown in Fig. 6, it becomes clear how NARIA can be employed to detect spatiotemporal data gaps and how it can be used to set future research agendas.

*NARIA* will be regularly updated to incorporate newly published data and any necessary amendments. Updated datasets will receive new DOIs and the old versions will continue to be stored within the repository for transparency. *NARIA* is a collaborative database that encourages and actively seeks new partnerships, data contributions, or data amendments from independent researchers and research groups to advance isotopic research in North American archaeology.

## Supplementary information

Supplementary Information

## Data Availability

The *North American Repository for Archaeological Isotopes (NARIA)* data community is available on the Pandora platform and is a member of the IsoMemo network of isotopic databases. The storage of this data is managed by the Max Planck Computing and Data Facility. More information on the NARIA Pandora data community can be found here: https://pandoradata.earth/organization/naria. The methods used to generate application examples presented in the Usage Notes section are described in Supplementary Text 1 and the codes employed are available at https://github.com/Pandora-IsoMemo/DSSM.
