# Supplementary material for: The North American Repository for Archaeological Isotopes
Source: Sci Data. 2025 Jan 11;12:50. doi: 10.1038/s41597-024-04175-2 (PMC11724993; doi:10.1038/s41597-024-04175-2)
Supplement: Supplementary file 1 — Supplementary Information [file 41597_2024_4175_MOESM1_ESM.docx]

Supplementary Materials for

**The North American Repository for Archaeological Isotopes**

Billings et al.

corresponding author(s): Traci N. Billings ([billings@gea.mpg.de](mailto:billings@gea.mpg.de)) & Ricardo Fernandes ([fernandes@gea.mpg.de](mailto:fernandes@gea.mpg.de))

This PDF file includes:

Tables S1 to S12

Text S1

References

**Metadata structure (adapted from the IsoMemo data entry forms)**

**Supplementary Table 1:** A description of the Structured Query Language (SQL) used to define data types of cells in Excel for the dataset framework.

| **SQL Data Type:** | **Description:** |
| --- | --- |
| Float | Approximate numeric type, from -1.79E+308 through 1.79E+308 |
| Small integer (smallint) | Exact numeric type, 2^15 (-32.768) through 2^15-1(32767) |
| Text | Character type, max 2^31-1 characters |
| Variable Character (varchar) | Character type, variable length, max 8000 characters |

**Supplementary Table 2:** Description of sample identifiers metadata from human and animal sources. *For animal sources only one column for ‘Age category individual’ is reported.

| **Column/Field** | **Data Type** | **Description** |
| --- | --- | --- |
| Sample ID | text | Sample code identification as given in publication |
| Individual ID | text | ID given to individual animals, humans, or plants. Allows us to connect multiple measurements made on the same individual reported in the same publication |
| Sample description | varchar | Other information related to the sample (e.g., cremated bone, preservation status, replicate, etc...) |
| Taxon | varchar | Taxonomic unit- genus/species name |
| Taxon family name | varchar | Taxonomic unit- family name |
| Common name | varchar | Common name of sample |
| General category | varchar | General animal category of the sample: amphibian, bird, cartilaginous fish, clam (Bivalvia), fish, mammal, mussel (Bivalvia), oyster (Bivalvia), ray finned fish, reptile, scallop (Bivalvia), shark cartilaginous fish, snail (Gastropoda) |
| Sex | varchar | Sex of animal/human: male (M); probable male (M?); female (F); probable female (F?); reported as indeterminate (I); not reported (-blank-) |
| Curated age category individual* | varchar | Age categories for humans: fetus (before birth), infant (0-3 years), child (3-12 years), adolescent (12-20 years), young adult (20-35 years), middle adult (35-50 years), old adult (50+ years), adult (over 20 but upper limit not determined) |
| Assigned age category | varchar | “x” if the reported age category of the individual was changed to fit the age categories in the *Curated age category individual column* |
| Reported age category | varchar | Age categories for humans as reported in original publication |
| Min. age individual (yrs) | float | Age lower limit of individual in years. If less than 1 year, then provided decimal value (e.g., 6 months = 0.5 year) |
| Max. age individual (yrs) | float | Age upper limit of individual in years. If less than 1 year, then provided a decimal value (e.g., 6 months = 0.5 year) |
| Osteological sample | varchar | Bone (e.g., femur, tibia, radius, vertebrae, etc...), Tooth (molar 1 (M1), molar 2 (M2), molar 3 (M3), premolar 1 (PM1), premolar 2 (PM2) canine (C), incisor 1 (I1), incisor 2 (I2), deciduous (d)) |
| Osteological sample part | varchar | Osteological part that was sampled (e.g., shaft, spinous process, distal portion, etc...) |

**Supplementary Table 3:** Description of sample identifiers metadata from plant sources.

| **Column/Field** | **Data type** | **Description** |
| --- | --- | --- |
| Sample ID | varchar | Sample code identification as given in the publication |
| Taxon (Order) | varchar | Taxonomic unit, Order (e.g., Flowering plant (angiosperms, monocot **OR** eudicot), Conifer (coniferophyta, pinales), etc…) |
| Taxon (Family) | varchar | Taxonomic unit, Family |
| Taxon (Genus Species) | varchar | Taxonomic unit, Genus, species |
| Common name | varchar | Common name of sample (e.g., maize, yucca, etc...) |
| General category | varchar | Common name of the general plant category to which the sample is assigned (cereal, weed, etc...) |
| Environment | varchar | If known, type of habitat plant sample is associated with (e.g., terrestrial, wetland, marine, etc…) |
| Photosynthesis Pathway | varchar | Does the plant use C_3_, C_4_, or CAM? |
| Plant part | varchar | Part of plant (e.g., kernel, seed, leaf, stem, etc...) from where the sample was taken |
| Fraction | varchar | Biochemical fraction for measurement |

**Supplementary Table 4:** Description of site information metadata.

| **Column/Field** | **Data Type** | **Description** |
| --- | --- | --- |
| Site name | varchar | Name of site where sample was recovered |
| Site description | text | Description of the site (e. g., cemetery, settlement, etc.) |
| Context description | text | Description of the context from where the sample was taken (e.g., single grave, collective grave, indications of status, etc...) |
| Context ID | varchar | Feature numbers, grave numbers, or any other specific information that helps to identify the context from where the sample was recovered |
| Altitude (MASL) | float | Altitude of site in meters (m) above mean sea level. |
| Latitude | float | Latitude (map datum WGS84) in decimal form |
| Longitude | float | Longitude (map datum WGS84) in decimal form |
| Exact Site location | yes/no | Is the exact site location known? |
| Radius (km) | float | If the specific location where the sample was recovered is not given, then a range can be defined. This field may also be useful if the sample came from an individual that was mobile and is not able to be assigned to a specific point or a specific location. |

**Supplementary Table 5:** Description of sample dating information metadata.

| **Column/Field** | **Data Type** | **Description** |
| --- | --- | --- |
| Min year (BCE/CE) | float | Date of the sample (lower limit of a 95% confidence interval). Dates before year 1 are represented as negative numbers. Reported as BCE/CE, if these dates are from radiocarbon they should be calibrated. |
| Max year (BCE/CE) | float | Date of the sample (upper limit of a 95% confidence interval). Dates before year 1 are represented as negative numbers. Reported as BCE/CE, if these dates are from radiocarbon they should be calibrated. |
| Dating method | varchar | Description of method(s) employed to date the sample, including any modelling. There are a variety of dating methods for the sample or context (e.g., artefact typology, epigraphy, radiocarbon dating following calibration, dendrochronology, etc...). |
| ^14^C | float | Radiocarbon age of sample (if available) as provided by radiocarbon dating lab- not modelled or corrected for any reservoir effect (e.g., uncal years before present (BP), 700+/-40 BP, 700 would go in this column). |
| ^14^C unc | float | Uncertainty of radiocarbon age of sample (if available) as provided by radiocarbon dating lab- not modelled or corrected for any reservoir effect (e.g., +/- years, 700+/-40 BP, 40 would go in this column). |
| Correction | float | Brief description if correction was added to the date (e.g., marine reservoir correction, local freshwater reservoir correction, etc…) |
| Period tags | text | Historical period tags separated by a semicolon (e.g., Archaic; Woodland; etc…) |

**Supplementary Table 6:** Description of δ^13^C & δ^15^N bulk collagen isotope measurements metadata.

| **Column/Field** | **Data Type** | **Description** |
| --- | --- | --- |
| Measurement ID collagen δ^13^C & δ^15^N | varchar | Multiple measurements may be performed on the same sample. This field serves to distinguish between measurements. |
| Lab name collagen δ^13^C & δ^15^N | varchar | Name of laboratory where measurement was taken |
| Nr. of measured samples (collagen δ^13^C & δ^15^N) | smallint | If the measurements reported correspond to an average of multiple measurements (resulting from separate pre-treatments or measurements made at different labs) their number is reported here. This is not the case if these are replicate measurements done on the same pretreated material in the same lab. |
| Sample preparation collagen δ^13^C & δ^15^N | text | Brief description of sample preparation and pretreatment |
| Analysis collagen δ^13^C & δ^15^N | text | Brief description of analytical parameters and instruments |
| IRMS collagen δ ^13^C | float | δ ^13^C/^12^C measurement in bone collagen. AMS ^13^C values should not be provided here. |
| IRMS collagen δ ^13^C unc. | float | Uncertainty of δ ^13^C/^12^C measurement in bone collagen. This is a combined uncertainty typically determined from replicate measurements. |
| Collagen δ ^15^N | float | δ ^15^N/^14^N measurement in bone collagen |
| Collagen δ ^15^N unc. | float | Uncertainty of δ ^15^N/^14^N measurement in bone collagen. This is a combined uncertainty typically determined from replicate measurements. |
| Collagen yield | float | Yield from collagen extraction |
| %C | float | C elemental concentration in bone collagen |
| %N | float | N elemental concentration in bone collagen |
| Atomic C:N ratio | float | C/N elemental atomic ratio |

**Supplementary Table 7:** Description of δ^13^C & δ^18^O bioapatite carbonate isotope measurements metadata.

| **Column/Field** | **Data Type** | **Description** |
| --- | --- | --- |
| Measurement ID carbonate δ^13^C & δ^18^O | varchar | Multiple measurements may be performed on the same sample. This field serves to distinguish between measurements. |
| Lab name δ^13^C & δ^18^O | varchar | Name of laboratory where measurement was taken |
| Nr. of measured samples (carbonate δ^13^C & δ^18^O) | smallint | If the measurements reported correspond to an average of multiple measurements (resulting from separate pre-treatments or measurements made at different labs) their number is reported here. This is not the case if these are replicate measurements done on the same pretreated material in the same lab. |
| Sample preparation carbonate δ^13^C & δ^18^O | text | Brief description of sample preparation and pretreatment |
| Analysis carbonate δ^13^C & δ^18^O | text | Brief description of analytical parameters and instruments |
| Carbonate δ^13^C | float | δ ^13^C/^12^C measurement in bone bioapatite carbonate |
| Carbonate δ^13^C unc. | float | Uncertainty of δ ^13^C/^12^C measurement in bone bioapatite carbonate. This is a combined uncertainty typically determined from replicate measurements. |
| Carbonate δ ^18^O | float | δ ^18^O/^16^O measurement in bone bioapatite carbonate |
| Carbonate δ ^18^O unc. | float | Uncertainty of δ ^18^O/^16^O measurement in bone bioapatite carbonate. This is a combined uncertainty typically determined from replicate measurements. |
| Reporting standard for Carbonate δ^18^O | varchar | Standard relative to carbonate δ^18^O measurement |

**Supplementary Table 8:** Description of δ^18^O bioapatite phosphate isotope measurements metadata.

| **Column/Field** | **Data Type** | **Description** |
| --- | --- | --- |
| Measurement ID phosphate δ^18^O | varchar | Multiple measurements may be performed on the same sample. This field serves to distinguish between measurements. |
| Lab name phosphate δ^18^O | varchar | Name of laboratory where measurement was taken |
| Nr. of measured samples (phosphate δ^18^O) | smallint | If the measurements reported correspond to an average of multiple measurements (resulting from separate pre-treatments or measurements made at different labs) their number is reported here. This is not the case if these are replicate measurements done on the same pretreated material in the same lab. |
| Sample preparation phosphate δ^18^O | text | Brief description of sample preparation and pretreatment |
| Analysis phosphate δ^18^O | text | Brief description of analytical parameters and instruments |
| Phosphate δ ^18^O | float | δ ^18^O measurement in bioapatite phosphate |
| Phosphate δ ^18^O unc. | float | Uncertainty of δ ^18^O measurement in bioapatite phosphate. This is a combined uncertainty typically determined from replicate measurements. |
| Reporting standard for Phosphate δ^18^O | varchar | Standard relative to phosphate δ^18^O measurement |
| Nr of meas. std. O | smallint | Number of analyses of quality control standard |
| δ ^18^O phos standard | float | δ ^18^O measurement in quality control standard |
| δ ^18^O phos unc standard | float | Uncertainty of δ ^18^O measurement in quality control standard |

**Supplementary Table 9:** Description of δ^87/86^ Sr bioapatite stable isotope measurements metadata.

| **Column/Field** | **Data Type** | **Description** |
| --- | --- | --- |
| Measurement ID ^87^Sr/^86^Sr | varchar | Multiple measurements may be performed on the same sample. This field serves to distinguish between measurements. |
| Lab name ^87^Sr/^86^Sr | varchar | Name of laboratory where measurement was taken |
| Nr. of measurements ^87^Sr/^86^Sr | smallint | If the measurements reported correspond to an average of multiple measurements (resulting from separate pre-treatments or measurements made at different labs) their number is reported here. This is not the case if these are replicate measurements done on the same pretreated material in the same lab. |
| Sample preparation ^87^Sr/^86^Sr | text | Brief description of sample preparation and pretreatment |
| Analysis ^87^Sr/^86^Sr | text | Brief description of analytical parameters and instruments |
| ^87^Sr/^86^Sr | float | δ ^87^Sr/^86^Sr measurement in bone bioapatite |
| ^87^Sr/^86^Sr unc. | float | Uncertainty of δ ^87^Sr/^86^Sr measurement in bone bioapatite. This is a combined uncertainty typically determined from replicate measurements. |
| Sr ppm | smallint | Concentration of Sr in parts per million (ppm) |

**Description of faunal data**

**Supplementary Table 10:** The number of isotopic measurements per taxon (family name) that have been currently compiled in the database.

| Accipitridae | 26 | Columbidae | 1 | Lutjanidae | 1 | Procyonidae | 110 |
| --- | --- | --- | --- | --- | --- | --- | --- |
| Acipenseridae | 15 | Corvidae | 1 | Mactridae | 6 | Psittacidae | 30 |
| Aetobatidae | 1 | Cottidae | 12 | Mammutidae | 15 | Ranidae | 5 |
| Alcidae | 18 | Cricetidae | 114 | Melongenidae | 3 | Salmonidae | 73 |
| Alligatoridae | 3 | Cyprindidae | 3 | Mephitidae | 4 | Sciaenidae | 11 |
| Amiidae | 2 | Delphinidae | 2 | Monodontidae | 3 | Sciuridae | 307 |
| Anatidae | 26 | Didelphidae | 8 | Mugilidae | 1 | Sebastidae | 75 |
| Antilocapridae | 49 | Diomedeidai | 1 | Muridae | 1 | Soricidae | 2 |
| Apocynaceae | 3 | Elephantidae | 39 | Mustelidae | 93 | Sparidae | 2 |
| Ariidae | 5 | Emydidae | 24 | Mylodontidae | 10 | Squalidae | 3 |
| Balaenidae | 7 | Equidae | 253 | Mytilidae | 289 | Stercorariidae | 1 |
| Bovidae | 771 | Erethizontidae | 5 | Naticidae | 6 | Stichaeidae | 2 |
| Camelidae | 75 | Esocidae | 1 | Noetiidae | 2 | Strigidae | 1 |
| Canidae | 774 | Fasciolariidae | 6 | Odobenidae | 8 | Suidae | 155 |
| Carcharhinidae | 1 | Felidae | 112 | Osmeridae | 6 | Tayassuidae | 1 |
| Cardiidae | 2 | Gadidae | 17 | Ostreidae | 3 | Tellinidae | 191 |
| Castoridae | 27 | Gaviidae | 1 | Otariidae | 31 | Testudine | 2 |
| Cathartidae | 5 | Geomyidae | 37 | Paralichthyidae | 1 | Tetraoninae | 1 |
| Catostomidae | 6 | Gomphotheriidae | 2 | Pectinidae | 2 | Trionychidae | 6 |
| Centrarchidae | 44 | Hexagrammidae | 10 | Percidae | 1 | Unionidae | 6 |
| Cervidae | 879 | Hystricomorph | 4 | Phasianidae | 610 | Ursidae | 88 |
| Cheloniidae | 1 | Ictaluridae | 4 | Phocidae | 70 | Valloniidae | 96 |
| Chimaeridae | 2 | Lepisosteidae | 21 | Phocoenidae | 10 | Veneridae | 5 |
| Clariidae | 21 | Leporidae | 567 | Pleuronectidae | 6 | Unknown | 92 |
| Clupeidae | 3 | Lotidae | 2 |  |  |  |  |

**Supplementary Text 1: Description of methods for modelled examples**

The research potential of NARIA is briefly exemplified in a data descriptor paper using two modeling examples (Figures 6-7). These utilise R-based^1^ modelling tools from the Pandora & IsoMemo initiatives - specifically TimeR, AverageR, and KernelTimeR - to investigate spatiotemporal trends in human-dog relationships and spatial mobility patterns in ancient North American populations. These modelling tools are detailed in Cocozza et al. 2022^2^ and can be accessed online via Shiny^3^ graphical interfaces (<https://isomemoapp.com/>) or run locally (code available on GitHub: <https://github.com/Pandora-IsoMemo/iso-app>).

For the human-dog relationship example, data potentially affected by diagenesis or poor measurements were excluded. This was done by filtering out records that did not fall within the established atomic C:N ratio criteria of 2.9-3.6^4^. Likewise, we removed data from human records that did not report collagen quality information, whenever these measurements were below -23‰ for δ¹³C or 5‰ for δ¹⁵N, assuming that they are unlikely to reflect well-preserved human collagen. To rule out a bias associated with potentially differentiated childhood diets, we excluded tooth samples and petrous bones from adult individuals, as these tissues provide a juvenile isotopic signal^5,6^, and non-adult measurements. Furthermore, measurements spanning more than 500 chronological years were discarded to enhance temporal precision and only data from domesticated dogs were included. Using the KernelTimeR tool - a 3-dimensional spatiotemporal kernel density estimator^7^ - we identified spatiotemporal clusters and locations that showed the best spatiotemporal distribution of dog data, which were then employed in the TimeR tool to explore temporal variation in isotopic data. TimeR is a Bayesian geostatistical model that estimates the expected value of a “dependent” variable across time and space.

For the spatial mobility pattern example, we generated a water δ^18^O baseline using the Cluster-based Water Isotope Prediction Model (RCWIP)^8^ in the AverageR tool. The mobility status of human individuals was determined by comparing their water δ^18^O values with the run model's baseline. Only permanent third molars’ enamel δ^18^O values were considered to avoid the potential interference of a breastfeeding signal in other teeth and consequent δ^18^O offsets^9^. The tooth enamel carbonate values, reported relative to the VPDB standard, were converted into values relative to the VSMOW standard (δ^18^O_VSMOW_ = δ^18^O_VPDB_ * 1.03092 + 30.92). These were then converted into phosphate values using the equation provided by Chenery et al. 2012 (δ^18^O_phosphate_ = δ^18^O_carbonate_ *1.0322 - 9.6849)^10^, and finally into water values following the methodology of Pollard et al. (2011) (δ^18^O_Water_ = δ^18^O_phosphate_ * 1.55 - 33.49)^11^. Individuals were categorized as mobile if their δ^18^O values fell outside the established 2-sigma credibility interval baseline range to account for known variability due to various factors such as diagenesis and cooking^12^.

**References**

1. R Core Team. R: A Language and Environment for Statistical Computing. R Foundation for Statistical Computing (2021).

2. Cocozza, C., Cirelli, E., Groß, M., Teegen, W.-R. & Fernandes, R. Presenting the Compendium Isotoporum Medii Aevi, a Multi-Isotope Database for Medieval Europe. *Sci Data* **9**, 354 (2022).

3. Chang, W., Cheng, J., Allaire, J., Xie, Y. & McPherson, J. Shiny: web application framework for R. *R package version* **1**, 2017 (2017).

4. DeNiro, M. J. Postmortem preservation and alteration of in vivo bone collagen isotope ratios in relation to palaeodietary reconstruction. *Nature* **317**, 806–809 (1985).

5. Hillson, S. *Teeth*. (Cambridge University Press, New York, 2005).

6. Jørkov, M. L. S., Heinemeier, J. & Lynnerup, N. The petrous bone-A new sampling site for identifying early dietary patterns in stable isotopic studies. *Am. J. Phys. Anthropol.* **138**, 199–209 (2009).

7. Wand, M. P. & Jones, M. C. Multivariate plug-in bandwidth selection. *Computational Statistics* **9**, 97–116 (1994).

8. Terzer, S., Wassenaar, L. I., Araguás-Araguás, L. J. & Aggarwal, P. K. Global isoscapes for δ18O and δ2H in precipitation: improved prediction using regionalized climatic regression models. *Hydrology and Earth System Sciences* **17**, 4713–4728 (2013).

9. Roberts, S. B. *et al.* Effect of weaning on accuracy of doubly labeled water method in infants. *American Journal of Physiology-Regulatory, Integrative and Comparative Physiology* **254**, R622–R627 (1988).

10. Chenery, C. A., Pashley, V., Lamb, A. L., Sloane, H. J. & Evans, J. A. The oxygen isotope relationship between the phosphate and structural carbonate fractions of human bioapatite. *Rapid Communications in Mass Spectrometry* **26**, 309–319 (2012).

11. Pollard, A. M., Pellegrini, M. & Lee-Thorp, J. A. Technical note: Some observations on the conversion of dental enamel δ18Op values to δ18Ow to determine human mobility. *American Journal of Physical Anthropology* **145**, 499–504 (2011).

12. Lightfoot, E. & O’Connell, T. C. On the Use of Biomineral Oxygen Isotope Data to Identify Human Migrants in the Archaeological Record: Intra-Sample Variation, Statistical Methods and Geographical Considerations. *PLOS ONE* **11**, e0153850 (2016).
